# Outlines of Chemistry for the Use of Students

**Published:** 1852-01

**Authors:** 


### REVIEWS.

---

Art. IV.—*Outlines of Chemistry for the Use of Students.* By William Gregory, M. D., Professor of Chemistry in the University of Edinburgh. First American, from the Second London Edition. Revised, corrected, and enlarged by J. Milton Sanders, M. D., L. L. D., Professor of Chemistry, Pharmacy, and Toxicology, in the E. Medical Institute of Cincinnati; and Professor of Natural Philosophy and Chemistry in the Literary Department of the Memphis Institute, etc. Cincinnati: H. W. Derby & Co., Publishers: 1851. 12 mo. pp. 614.

We have copied the title page to this book in full, because it is an epitome of the work, and a token of the high claims of its American editor.

Dr. Gregory is the best English exponent of the continental, and especially of the Giessen school of chemists He is at once the organ and the echo, of the illustrious Liebig, by whose word he swears. We read his outlines of Liebig's organic chemistry in 1845, with great pleasure and profit. It was the first English dress of the compound radical theory carried out in all the detail of this most prolific and changing science, a lucid but inaccurately printed digest of the great treatise on organic chemistry by Liebig, the French issue of which is better known to chemists in America than the original German. Since then Dr. Gregory has added to his outlines of organic chemistry, the inorganic portion of the science, and in 1847, the two appeared united in the present form.

The distinguished successor of the renowned and lamented Hope needs no praise from our hands. Nor do we propose to sound his praises, or criticise his labors. It is enough that he is the chosen organ of the school of Liebig, and the author of an excellent elementary treatise, with which all teachers of chemistry are entirely familiar. This treatise is worthy of remark in one important particular, in which it differs from most others which have appeared before in England or America. We allude to the omission in its pages of all allusion to what are familiarly called the "imponderables"—the *physique* of chemistry. Where a chemist can teach a class of students who are all graduates of a college, where the general principles of physics are given, in the same way in which Geometry, Algebra, and the Natural Sciences are taught, as a part of elementary instruction, it is a great relief to him to have no necessity of teaching the doctrines of heat, or the principles of electricity, in its various departments. But it is not our experience in this country, and especially in our medical schools, that the classes who assemble to hear chemical lectures have this previous preparation. Too commonly their first intellectual efforts, in scientific acquirements, are made under the oral guidance of their teachers, whose arduous duty it is to break up for the first time, the fallow ground of uncultivated minds.—With such pupils, some time is unavoidably bestowed on those topics which are not the less indispensable, because they are more properly preliminary, and without which subsequent progress must be always halting and intricate For these reasons, and others which we could give were it required, we doubt whether Dr. Gregory's book with all its excellent points will meet the wants of *students*, and especially *medical students* in this country. To the teacher it must always be a useful laboratory book, and especially because of the fullness of the organic portion.

Dr. Gregory in his preface tells us that he gives three and a half out of five and a half months of his course to organic chemistry, and regards this as a full compensation to the student, for the absence of the chapters on light, heat, electricity, &c., and the comparative meagreness of those on the inorganic and metallic elements.—This may be, and probably is, exactly what is required in the high-toned University of Edinburgh, where a great majority of the students are advanced scholars, and literary graduates. But it is unsuited to the purposes of American teachers; most of whom must content themselves with such instruction as they can crowd into courses of lectures, few of which reach the number allotted as the years of human life, and still fewer exceed that limit.

But we have not taken up our pen to write an essay on the present method of chemical instruction in this country, the faults of which we appreciate, however unable we may be to correct them. All that we have said or may say regarding the excellencies of the work in question is applicable solely to the original work, and not to the edition which has been "revised, corrected, and enlarged, by J. Milton Sanders, M. D., L. L. D."

That Dr. Gregory should have sought an American editor for his "Outlines" in the "E. Medical Institute of Cincinnati," may well excite our surprise, but we are assured by the learned Doctor of Medicine and of Laws, that he willingly undertakes "at the flattering request of Prof. Gregory to edit this American edition of his excellent work, adding such notes as the late progress of the science requires." We might quote farther from the American preface how much this edition is superior in accuracy to the London one, and how the notes which have been added render this more valuable than the English edition. We are warranted then to believe, that the reader will find this edition fully posted in all that is new or important in the science, and we anticipate with pleasure, the profit we are to derive from those "*notes*," which have given to the American edition a value not found in the English.—What is our disappointment then at finding all the discoveries of the past four or five years comprised in the limits of fifteen foot notes, for the most part singularly insignificant, and amounting in all to less than a page and a half of fine print! We then turn with avidity to the table of equivalents to note there the progress of our favorite science, as posted up in this significant balance-sheet of the results of chemical research. We had heard that several important changes had been made in the atomic weights of some well known substances. We had heard, for example, that bismuth had been determined anew by Schneider, and set down at 208;—that tungsten had the number 92; glucinum 4.7; gold 197; uranium 60; and so on. But we presume that the journals and annual reports of progress are wrong on these points, for we find that Dr. Sanders, L. L. D., permits the old numbers to stand unchanged. We had also gladly accepted the rumor that the views of Dr. Prout were rapidly finding confirmation in a greatly increased number of substances, which had been found to possess an atomic weight, the multiple of hydrogen, and we were consoling ourselves, that we might count arsenic, antimony, bromine, cadmium, calcium, carbon, cerium, gold, iodine, iridium, mercury, molybdenum, oxygen, phosphorus, sodium, sulphur, thorium, tin, titanium, tungsten, uranium, &c., as whole numbers, the simple multiples of hydrogen, and free from vexatious fractions. But since the fully "corrected, revised, and enlarged" edition of Dr. Gregory's Outlines emanates from the "E. Medical Institute," with the old numbers unchanged, we are convinced that Baron Liebig's annual report, and even the American scientific journals are quite wrong and hasty in making such assertions. What a delightful thing it is to have some great name to lean upon again, now that poor Berzelius is no more! Since the death of the great Swede, our faith, we confess, has been somewhat shaken in these old numbers, so laboriously determined by him, and which we learned in our youth; but now, this new Berzelius of the West reassures our doubting minds, and strengthens our conservatism. We do not, however, quite comprehend the meaning of the note on page 38, about doubling the equivalents of antimony, arsenic, sulphur, and phosphorus, since the text was written, because in the table of equivalents in the text, we find sulphur and phosphorus double already, though why we do not understand, as we say, in view of the note; and we are under the impression that Dr. Gregory is alone in doubling the number of sulphur, which all now agree upon as correctly stated to be 16. On turning to the article sulphur, we find that substance treated there under the old atomic weight, thus showing a discrepancy in the work itself, which if it did not originate with the American editor, ought to have been corrected by him.

We had also heard much of the remarkable researches of Hofmann and Wurtz, the former on the molecular structure of organic bases, and the latter on alkaloids homologous with ammonia; as constituting the great discoveries of this decade in organic chemistry. But our American editor passes them in silence, and regales us, instead, with a brief notice appended to "spirit varnishes" of recent investigations at the Memphis Institute, upon the active principles of several medicinal plants; '*resinoids*,' which have been found highly valuable remedies, and at present extensively used; and the medicinal properties of these '*resinoids*' are believed to lie in more active principles, analogous to the alkaloids in nux vomica, opium, &c. (p. 474.)

But to speak seriously and in terms not to be misunderstood, we declare that, with a few unimportant exceptions in the *fifteen notes* alluded to before, the whole field of organic and inorganic chemistry remains ungleaned of the rich results of the past four years of remunerative and fruitful labor, so far as the editorial contributions of Prof. Sanders are concerned. Had the editor modestly stated that he had simply seen the sheets through the press, we should have been saved the trouble of this notice, however we might have regretted the numerous typographical errors with which it is disfigured.

